# Copper homeostasis and copper-induced cell death in the pathogenesis of cardiovascular disease and therapeutic strategies

**DOI:** 10.1038/s41419-023-05639-w

**Published:** 2023-02-11

**Authors:** Xinyue Chen, Qi Cai, Ruikai Liang, Deju Zhang, Xiao Liu, Meiying Zhang, Yan Xiong, Minxuan Xu, Qi Liu, Pengyang Li, Peng Yu, Ao Shi

**Affiliations:** 1 The Second Clinical Medical College of Nanchang University, The Second Affiliated Hospital of Nanchang University, Nanchang, Jiangxi China; 2 Food and Nutritional Sciences, School of Biological Sciences, The University of Hong Kong, Pokfulam Road, Hong Kong, China; 3 Department of Cardiovascular Medicine, The Second Affiliated Hospital of Sun Yat-Sen University, Guangzhou, Guangdong China; 4 Department of Endocrinology and Metabolism, The Second Affiliated Hospital of Nanchang University, Nanchang, Jiangxi China; 5 Wafic Said Molecular Cardiology Research Laboratory, The Texas Heart Institute, Houston, TX USA; 6 Division of Cardiology, Pauley Heart Center, Virginia Commonwealth University, Richmond, VA USA; 7 Faculty of Medicine, St. George University of London, London, UK; 8 University of Nicosia Medical School, University of Nicosia, Nicosia, Cyprus

**Keywords:** Cell death, Cardiovascular diseases

## Abstract

Copper is a vital mineral, and an optimal amount of copper is required to support normal physiologic processes in various systems, including the cardiovascular system. Over the past few decades, copper-induced cell death, named cuproptosis, has become increasingly recognized as an important process mediating the pathogenesis and progression of cardiovascular disease (CVD), including atherosclerosis, stroke, ischemia-reperfusion injury, and heart failure. Therefore, an in-depth understanding of the regulatory mechanisms of cuproptosis in CVD may be useful for improving CVD management. Here, we review the relationship between copper homeostasis and cuproptosis-related pathways in CVD, as well as therapeutic strategies addressing copper-induced cell death in CVD.

## Facts

- Copper is an essential micronutrient that regulates a wide range of biologic processes.
- Cuproptosis, or copper-induced cell death, is believed to promote the pathogenesis of cardiovascular disease.
- Cardiovascular disease treatments targeting copper homeostasis have been carried out in recent years, although with certain limitations.

## Open questions

- What is the phenotype of copper-induced cell death?
- How does the aggregation of fatty-acylated proteins induce a cascade of cell killing?
- Are there other important roles for copper in mitochondria?
- How can the appropriate copper concentration be determined for the treatment of different types of cardiovascular disease?
- How can the performance of copper conditioners be optimized, and their defects reduced?

## Introduction

Cell death is accompanied by inflammatory dysregulation, cellular dysfunction, and tissue damage, and it plays a critical role in the progression and pathogenesis of cardiovascular disease (CVD) [1]. Over the past few decades, copper-induced cell death, named cuproptosis, has been widely reported in a variety of CVDs [2], including atherosclerosis [3], stroke [4], ischemia-reperfusion injury [5], and heart failure [6].

Copper (Cu) is a catalytic cofactor for multiple physiologic processes such as energy metabolism, mitochondrial respiration, and anti-oxidation [7]. In general, intracellular Cu concentration is relatively low. When Cu levels build up inside cells, excessive Cu ions bind to mitochondrial proteins, leading to proteotoxic stress-mediated cell death [8]. In CVD, cuproptosis disturbs lipid metabolism and contributes to oxidative stress, mitochondrial damage, and endothelial cell dysfunction [9–11]. In this review, we summarize the current literature focused on copper-induced cell death in CVD. Furthermore, we discuss potential therapeutic strategies involving copper-induced cell death and provide key insights into potential new clinical treatments for cuproptosis-related cardiac manifestations.

## Copper metabolism-related molecules and mechanisms in mammals

**Fig. 1:**
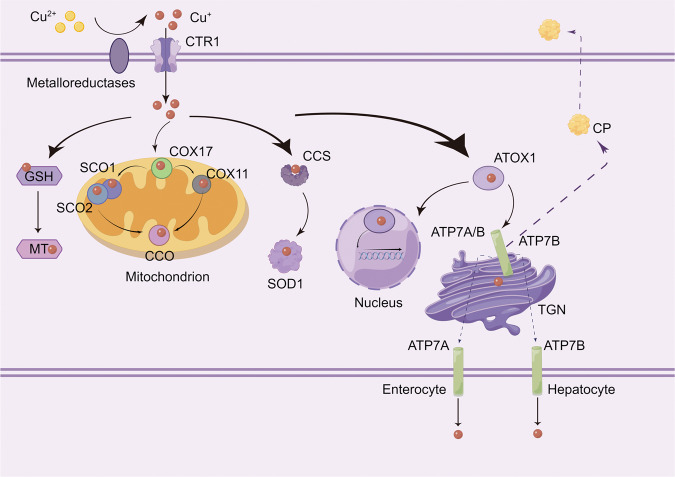
Copper metabolism in mammals at a molecular level. Cu^+^ can be sequestered by MT for storage. CTR1 is highly specific for the uptake of Cu^+^. At physiologic Cu^+^ levels, copper-transporting ATPases localize in the TGN, where they pump Cu^+^ from the cytoplasm into the lumen of the TGN. When intracellular Cu^+^ increases, these copper-transporting ATPases fuse with the plasma membrane to export Cu^+^. In the basolateral membrane of enterocytes, copper is pumped by ATP7A into the portal circulation and enters the main organ of copper storage, the liver. Excess copper in liver cells is secreted into bile in the form of vesicles via ATP7B. Cu^+^ travels through the copper transport ATP7B-TGN pathway to form CP, which is then transported to various systems throughout the body. In addition, ATOX1 transports Cu^+^ to the nucleus, where it binds to transcription factors and drives gene expression. COX17 transports Cu^+^ to the copper-carrying proteins SCO1, SCO2, and COX11 and delivers it to CCO to activate the activity of enzymes in the respiratory chain. CCS can transfer Cu^+^ to SOD1. ATOX1 antioxidant-1, ATP7A copper-transporting ATPase alpha, ATP7B copper-transporting ATPase beta, CCO cytochrome C oxidase, CTR1 copper transporter 1 of CCO, CCS Cu chaperone for SOD1, COX11 cytochrome c oxidase copper chaperone 11, COX17 cytochrome C oxidase copper chaperone 17, CP ceruloplasmin, GSH glutathione, MT metallothionein, SCO1 synthesis of cytochrome C oxidase 1, SCO2 synthesis of cytochrome C oxidase 2, SOD1 superoxide dismutase 1, TGN trans Golgi network. The figure was created with Figdraw (https://www.figdraw.com/).

Copper is an important dietary micronutrient. It has two different ionic forms: Cu^+^ (cuprous ion, reduced form) and Cu^2+^ (copper ion, oxidized form), which participate in the enzymatic regulation of cellular physiologic functions [12]. As shown in Fig. 1, before entering the cell, Cu^2+^ is absorbed on the cell surface by metalloreductases, such as six-transmembrane epithelial antigen of the prostate (STEAP), and reduced to Cu^+^. Then, copper uptake is mediated by copper transporter 1 (CTR1), which forms homotrimers on the intestinal epithelial cell membrane to specifically absorb Cu^+^ [13]. Once entering the circulation, copper is transported to organs and tissues by binding to plasma proteins such as ceruloplasmin (CP), albumin, trans copper protein, and other plasma proteins [14, 15]. Hepatocytes in the liver are the major storehouse for Cu [16]. Cu can be shuttled to protein targets by different protein carriers, such as the chaperone protein cytochrome C oxidase copper chaperone 17 (COX17) located in the cytoplasm and mitochondrial membrane space. COX17 transports Cu^+^ to secondary copper-carrying proteins including synthesis of cytochrome C oxidase 1 (SCO1), synthesis of cytochrome C oxidase 2 (SCO2), and cytochrome C oxidase copper chaperone 11 (COX11) and delivers Cu^+^ to the cytochrome C oxidase (CCO) II and I subunits to activate the activity of enzymes in the respiratory chain [17]. Copper chaperone for superoxide dismutase (CCS) is another Cu chaperone protein that transports Cu^+^ to participate in various physiologic processes such as oxidation, protein synthesis, and protein secretion [18]. CCS can transfer Cu^+^ to superoxide dismutase 1 (SOD1) and function in an anti-oxidative-stress role [19]. A third major copper chaperone protein is antioxidant-1 (ATOX1). ATOX1 can transport Cu^+^ to the nucleus, where it binds to transcription factors and drives gene expression. In addition, ATOX1 transfers Cu^+^ from the trans-Golgi network (TGN) to copper-transporting ATPase (ATP7 alpha [ATP7A] and ATP7 beta [ATP7B]) [20, 21]. ATP7A and ATP7B are expressed in a tissue-specific manner. ATP7A is expressed in most tissues and organs except the liver [21], whereas ATP7B is primarily expressed in the liver [21]. At physiologic Cu^+^ levels, copper-transporting ATPases localize in the TGN, where they pump Cu^+^ from the cytoplasm into the lumen of the TGN [22]. When intracellular Cu^+^ increases, these copper-transporting ATPases fuse with the plasma membrane to export Cu^+^. When copper levels return to physiologic levels, these copper transporters are recycled back to the TGN [23]. In Table 1, the principal modulators of copper metabolism are summarized.

**Table 1:** Principal modulators of copper metabolism.

Molecular	Function	Ref.
CTR1	Transport of copper as a transmembrane solute carrier	[132]
CP	Copper-containing enzyme protein, antioxidant	[133]
SOD1	Copper-containing enzyme protein, antioxidant metalloenzyme	[134]
ATOX1	Copper chaperone protein	[135]
ATP7A	Transport copper	[136]
ATP7B	Transport copper	[136]
STEAP	Metalloreductase, reduce copper	[137]

*ATOX1* antioxidant 1, *ATP7A* ATPase copper transporting alpha, *ATP7B* ATPase copper transporting beta, *CP* ceruloplasmin, *CTR1* copper transporter 1, *SOD1* superoxide dismutase 1, *STEAP* six-transmembrane epithelial antigen of the prostate.

From a cellular perspective, Cu contributes to different cellular activities. The extracellular divalent Cu^2+^ regulates the interaction between growth factors and cell membrane receptors [12]. Once it reaches cell membranes, Cu^2+^ will be reduced by metalloreductases. Then, monovalent Cu^+^ can modify protein structure or phosphorylation status, altering the activation status of growth factor receptors on the plasma membrane [12]. In the cytoplasm, Cu^+^ maintains the redox balance in various organelles and directly regulates kinase activity through the structural modification of phosphatase [12]. In the nucleus, Cu^+^ regulates gene expression and subsequent protein synthesis by binding to transcription factors [12].

## Copper-induced cell death

### Discovery of copper-induced cell death

In 1978, Chan et al. discovered the mechanisms that regulate intracellular copper in normal fibroblasts and that high concentrations of copper lead to cell death [24]. When the copper concentration in the medium of normal fibroblasts was higher than 30 μg/ml (the intracellular copper concentration was 19 times that of the basal medium), fibroblasts died [24]. However, the underlying mechanisms were unclear. Since then, the mechanism of copper-induced cell death has attracted the interest of researchers, and the dual role of copper ions has been revealed. When the homeostasis of copper ion is disrupted, this imbalance can trigger cytotoxicity and induce cell death through various pathways, including reactive oxygen species (ROS) accumulation, proteasome inhibition, and mitochondrial dysfunction. This newly identified mode of regulatory cell death has been named “cuproptosis.” During this process, copper ions bind to fatty-acylated proteins in the tricarboxylic acid cycle during mitochondrial respiration, resulting in fatty-acylation modification [8]. The protein aggregation of iron-sulfur clusterin in turn leads to the downregulation of iron-sulfur clusterin expression, which induces proteotoxic stress and eventually causes cell death [8]. However, the phenotype of copper-induced cell death and the regulatory mechanisms of its signaling cascade remain to be further explored. In the next section, we further elaborate on the molecular and metabolic mechanisms of copper-induced cell death in CVD.

### Crosstalk between copper-induced cell death and other cell death pathways

Over the past few decades, many studies have suggested that cuproptosis is closely associated with ROS and inflammation and that it triggers other forms of cell death including apoptosis, pyroptosis, and ferroptosis. Luo et al. treated mouse mononuclear macrophages with CuSO_4_ and found that mitochondrial ROS levels in the cells increased and induced apoptosis, whereas ROS inhibitors rescued cell viability [25]. Further studies identified the dependence of ROS-induced apoptosis on the persistent activation of pro-apoptotic mitogen-activated protein kinase (MAPK) pathways (cJun N-terminal kinases [JNKs] and p38), which modulated the phosphorylation of mitochondrial pro-apoptotic and anti-apoptotic proteins [26, 27]. Similarly, Yip et al. found that the disulfiram-Cu^2+^ complex induced the production of ROS, which in turn activated downstream apoptosis-related JNK and p38MAPK pathways, thereby inducing breast cancer cell apoptosis [28]. From an inflammation perspective, activation of the nucleotide-binding oligomerization domain, leucine-rich repeat and pyrin domain-containing protein 3 (NLRP3) inflammasome pathway was found to introduce copper-mediated macrophage pyroptosis, which is an inflammatory form of lytic programmed cell death [29]. Similar results were found in mouse macrophages treated with copper oxide nanoparticles (CuONPs), which showed an increased level of proinflammatory factors including NLRP3, caspase-1, and interleukin (IL)-1β [30]. In the acidic environment of lysosomes, CuONPs attacked lysosomes by releasing copper ions, resulting in the release of cathepsin B, which directly mediated the activation of the NLRP3 inflammasome [30]. Also, CuONP exposure triggered macrophages to express pro-IL-1β via activation of the myeloid differentiation factor 88 (MyD88)-dependent Toll-like receptor 4 (TLR4)/nuclear transcription factor kappa B (NF-κB) cascades, which is the canonical pathway of NLRP3 inflammasome activation [30, 31].

Another cuproptosis-connected form of cell death is ferroptosis, which is iron-dependent cell death characterized by the disruption of iron homeostasis and the accumulation of lipid ROS [32]. Ren et al. found that cellular mitochondrial homeostasis was disrupted, and that mitochondrial fragmentation occurred and gathered around the nucleus by accumulating copper ions in hepatocellular carcinoma cells [33]. They also observed an increase in free iron pools, superoxide, and lipid peroxides in cells. We speculate that copper synergistically induces cuproptosis and ferroptosis in hepatocellular carcinoma cells treated with anticancer drugs—an area that deserves further exploration. From the studies described above, we know that copper is closely related to apoptosis, pyroptosis, and ferroptosis. Also, cross-talk occurs between different forms of cell death and introduces a series of cell death events. In the future, uncovering the mechanisms linking several modes of cell death will be crucial. This will also provide new ideas for the combined use of therapeutic drugs targeting different modes of cell death.

## Mechanism of copper-induced cell death in CVD

### Oxidative stress

**Fig. 2:**
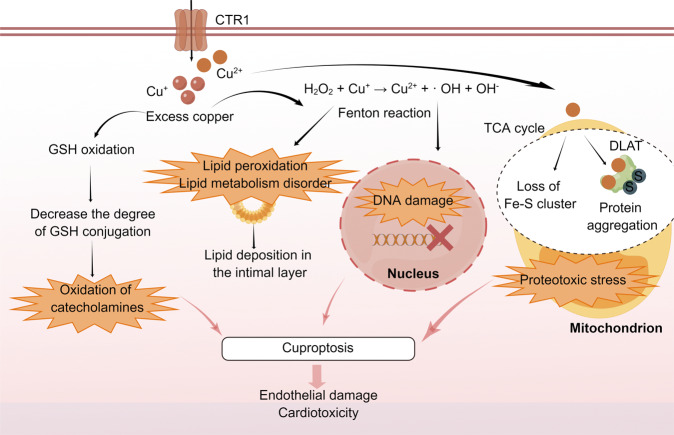
Oxidative stress induced by copper-induced cell death in CVD. Excess copper results in the oxidation of catecholamines by promoting GSH oxidation. Through the Fenton reaction, copper produces oxidative stress, increasing lipid metabolism dysfunction and leading to DNA breakage. Copper ions directly bind fatty acylation components in the TCA cycle, leading to the aggregation and dysregulation of these proteins, blocking the TCA cycle of the tricarboxylic acid cycle, triggering proteotoxic stress, and inducing cell death. The above mechanisms may lead to endothelial injury and cardiotoxicity. CVD cardiovascular disease, CTR1 calcitonin receptor 1, DLAT dihydrolipoamide S-acetyltransferase, Fe-S iron-sulfur proteins, GSH glutathione, S sulfur ion, TCA tricarboxylic acid cycle. The figure was created with Figdraw (https://www.figdraw.com/).

Cells maintain a balanced cycle of oxidation and anti-oxidation. When oxidative homeostasis is disturbed in the cardiovascular system, this leads to oxidative stress generation, resulting in related cell damage and the occurrence of CVD. The Fenton reaction is one of the most important metal-mediated reactions [34]. Copper ions cycle between oxidation and reduction states and form hydroxyl radicals [35]. Hydroxyl radicals react with DNA and lipids, causing DNA damage and lipid peroxidation, respectively. Moreover, oxidative stress caused by excess copper causes disorders of lipid metabolism, resulting in lipid deposition in the intimal layer that leads to atherosclerosis [36–38]. Li et al. showed that metabolite levels in pig cardiomyocytes changed after copper exposure and that the metabolite changes were mainly involved in glycerophospholipid metabolism and fatty acid extension, as well as degradation processes [39]. In addition, copper increased glutathione oxidation through oxidative stress and reduced the degree of glutathione conjugation [40], resulting in the oxidation of catecholamines in vivo and subsequent cardiotoxicity. Notably, a recent study identified copper-associated proteotoxic stress [8] that may also be involved in the pathogenesis of CVD. The mechanisms of oxidative distress discussed above are shown in Fig. 2.

### Mitochondria and copper in CVD

As the energy factory of eukaryotic cells, the mitochondria coordinate cellular metabolic processes such as oxidative phosphorylation. Micronutrients such as copper are essential for normal mitochondrial effects, especially in mitochondrial tissues such as the heart muscle. In mitochondria, Cu^+^ is an important component of complex IV, also known as CCO, which can activate enzyme activity in the respiratory chain [41]. Copper deficiency results in the decreased transport of copper to SCO1/SCO2 and COX11 via COX17, inducing decreased synthesis of CCO. In rats, a copper-deficient diet resulted in a 74% decrease in CCO [42]. Similarly, the decreased CCO activity due to copper deficiency was also reported by Johnson et al. [43]. In addition, copper deficiency can lead to mitochondrial dysfunction by inducing the expression of other mitochondria-related molecules. Peroxisome proliferator-activated receptor-gamma coactivator-1 alpha protein (PGC-1α) is a master regulator of mitochondrial biogenesis [44]. High expression of PGC-1α can interfere with the ultrastructure of mitochondria, leading to dysfunctional mitochondrial proliferation and myocardial disease [44]. Medeiros et al. showed that copper deficiency can cause myocardial dysfunction by increasing the expression of PGC-1α [45].

**Fig. 3:**
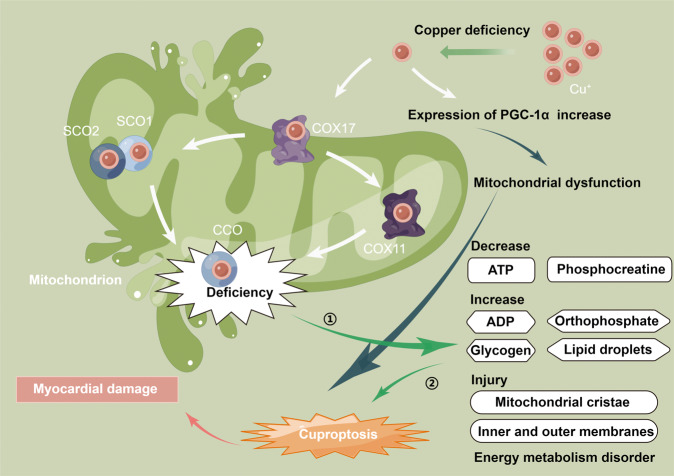
Mitochondria and copper in CVD. When copper deficiency reduces the activity of CCO, the level of ATP and phosphocreatine in the heart and other tissues decreases, whereas the content of ADP, orthophosphate, glycogen, and lipid droplets increases. Mitochondrial cristae and inner and outer membranes are altered, eventually leading to mitochondrial rupture. Additionally, copper deficiency increases the level of PGC-1α, causing mitochondrial dysfunction. These changes interfere with energy metabolism and cause myocardial damage. CVD cardiovascular disease, CCO cytochrome C oxidase, COX11 cytochrome c oxidase copper chaperone 11, COX17 cytochrome C oxidase copper chaperone 17, PGC-1α peroxisome proliferator-activated receptor-gamma coactivator-1 alpha protein, SCO1 synthesis of cytochrome C oxidase 1, SCO2 synthesis of cytochrome C oxidase 2. The figure was created with Figdraw (https://www.figdraw.com/).

The downregulated expression and activity of CCO cause stiff cardiomyocyte fibers, increased glycogen and lipid droplets, and dysfunctional cardiac tissue, resulting in fatal heart disease [42, 46]. ATP and phosphocreatine in the heart and other tissues decrease, while ADP and orthophosphate content increase. Simultaneously, mitochondrial cristae and inner and outer membranes are altered, eventually leading to mitochondrial rupture. These changes interfere with energy metabolism and cause myocardial damage. The above-described mechanisms of mitochondria and copper in CVD are shown in Fig. 3.

### Vascular regulation and copper

**Fig. 4:**
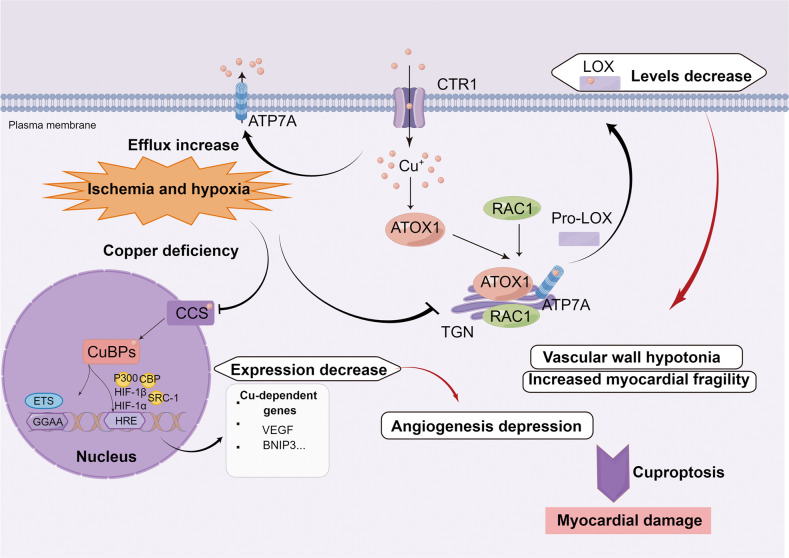
Vascular regulation and copper in CVD. Copper can regulate the activity of HIF-1. HIF-1 consists of HIF-1α and HIF-1β. CCS transports copper into the nucleus, and CuBP mediates subsequent actions of copper. The core base “GGAA” (the core motif of the ETS family) is a key motif in the binding site of copper-dependent genes. p300, CBP, and SRC1 act as cofactors to form the HIF-1 transcription complex. The interaction of HIF-1 with HRE requires copper to initiate the copper-dependent expression of genes such as VEGF. LOX is essential for vascular maturation. Copper can regulate LOX production through ATOX1, ATP7A, and RAC1. Ischemia and hypoxia increase the efflux of copper. The inhibition of the above mechanisms caused by copper efflux will bring about vascular wall hypotonia, increased myocardial fragility, and angiogenesis depression, and will eventually lead to myocardial damage. ATOX1 antioxidant 1 copper chaperone, ATP7A ATPase copper transporting alpha, BNIP3 BCL2 interacting protein 3, CCS Cu chaperone for SOD1, CTR1 calcitonin receptor 1, ETS E-twenty-six, HIF hypoxia inducible factor, HRE hypoxia-responsive element, LOX lysyl oxidase, RAC1 ras-related C3 botulinum toxin substrate 1, TGN trans Golgi network, VEGF vascular endothelial growth factor. The figure was created with Figdraw (https://www.figdraw.com/).

Hypoxia-inducible factor 1 (HIF-1) is a major transcription factor that regulates angiogenesis [47]. Cardiac copper concentration gradually decreases after ischemic injury and is positively correlated with HIF-1-mediated expression of angiogenic and glycolytic genes [48]. In prolonged myocardial infarction, a major factor regulating HIF-1 activity is HIF-1a, a key HIF-1 subunit. Multiple aspects of HIF-1 regulation are regulated by copper, including the stabilization of HIF-1a, the formation of transcription complexes, and the binding to the hypoxic response element (HRE) sequences of target genes [47]. CCS brings copper into the nucleus, and Cu-binding proteins (CuBPs) mediate the subsequent actions of copper. The core base “GGAA” (the core motif of the E-twenty-six [ETS] family) is a key motif in the binding site of copper-dependent genes. p300, CREB binding protein (CBP), and steroid receptor coactivator-1 (SRC-1) act as cofactors to form the HIF-1 transcription complex [47]. The interaction of HIF-1 with HRE requires copper to initiate the copper-dependent expression of genes such as *VEGF*, *BNIP3*, and other angiogenic genes [49, 50]. In addition, in recent years, lysine oxidase (LOX) has been found to severely affect cardiovascular function [51]. The CTR1/ATOX1/ATP7A/RAC1 pathway promotes the conversion of pro-LOX to LOX during the transportation of Cu^+^ [51]. LOX and LOX-like (LOXL) promote the cross-linking of elastin and matrix collagen. The lack of copper decreases the amount and activities of LOX, resulting in the degradation of collagen fibers and elastic fibers, and, in severe cases, rupture of the vascular intima [52]. The detailed process described above is shown in Fig. 4.

Together, these data have pointed to oxidative stress, mitochondrial damage, and vascular growth as the main mechanisms in CVD relevant to copper-induced cell death. In the next section, we discuss the role of copper homeostasis in atherosclerosis, stroke, ischemia-reperfusion injury, and heart failure, respectively.

## Copper homeostasis and CVD

### Atherosclerosis

Atherosclerosis is an inflammatory condition associated with lipid deposition in the intimal layer of the vessel walls, leading to occlusion of the blood vessel and promoting the development of CVD [53]. The unstable state of atheroma formation may promote plaque detachment and lead to wall damage [53]. Notably, the development of atherosclerosis is associated with previously introduced molecules involved in copper homeostasis. First, the accumulation and oxidation of excess low-density lipoprotein (LDL) cholesterol is considered central to the process of atherosclerosis [54]. The downregulation of ATP7A can attenuate the cell-mediated oxidation of LDL in THP-1 macrophages [55] and may reduce macrophage infiltration [56]. Meanwhile, ATOX1 and ATP7A are jointly involved in copper-induced vascular smooth muscle cell (VSMC) growth [52, 57]. VSMC migration is a key process in the development of remodeling after atherosclerosis and vascular injury [57, 58].

Copper also reduces the occurrence and development of atherosclerosis by inhibiting inflammation-related pathways. The Notch signaling pathway is critical in regulating chronic inflammation in atherosclerosis [59]. Zhao et al. found that Cu^2+^ coordination polymer inhibited the Notch signaling pathway, significantly reducing the inflammatory events in atherosclerotic segments [60]. Also, Wang et al. found that copper supplements could inhibit atherosclerotic lesions by reducing endothelial cell mortality, lowering cholesterol and phospholipid concentrations in lesion tissue, and minimizing atherosclerotic lesion size [3]. It is worth noting that a reduction in copper in atherosclerotic lesions is associated with an increase in serum copper concentrations [61]. However, the cause of copper reduction in atherosclerotic walls is unknown and needs to be further explored. Optimal concentrations of copper supplementation decrease atherosclerosis, whereas dietary copper deficiency or excess is associated with increased susceptibility to aortic atherosclerosis. Lamb et al. found that the optimal dietary copper intake (1–3 mg/day) was associated with a decrease in susceptibility to aortic atherosclerosis, as shown in rabbits fed with cholesterol compared with rabbits fed deficient diets (0 mg/day) and high copper supplementation (20 mg/day) [62].

Notably, Koksal et al. found that cellular copper levels are higher in pathologic inflammatory conditions such as atherosclerosis [63], which is contrary to the research described above. Given that o-tyrosine, a marker of copper-induced protein oxidation, is detected only in advanced human atherosclerotic lesions rather than in the early stages of atherosclerosis [64], we speculate that copper level is increased only in advanced atherosclerotic tissues. Additionally, Bügel et al. found that increasing copper intake can reduce the risk of human atherosclerosis [65], which suggests that the concentration of copper in the lesions of patients with early atherosclerosis may be low. Moreover, Tasić et al. found a difference in copper content between calcified and fibrolipid plaques in patients with carotid atherosclerosis, whereby the copper content was lower in fibrolipid plaques than in calcified plaques [66]. Thus, we speculate that differences in copper concentrations in human atherosclerotic tissues may reflect not only the stage, but also the context of atherosclerotic lesions. Interestingly, Diaf et al. found no significant association between dietary copper intake and atherosclerosis risk in patients with diabetes, but the highest quarter of copper intake was associated with the risk of atherosclerosis in patients without diabetes [67]. Therefore, the level of copper in different stages of atherosclerosis progression is also affected by other comorbidities. These differences will affect the choice of clinical treatment and warrant further studies.

### Stroke

Globally, stroke is the second leading cause of death [68]. In a meta-analysis of eight studies including 777 participants, investigators found that serum copper levels were significantly higher in the ischemic stroke group than in the control group [69]. However, another case-control study based on the 2013 to 2018 National Health and Nutrition Examination Survey showed that stroke risk and dietary copper intake are inversely related [70]. On the other hand, in another clinical study, the serum copper level was shown to be significantly lower in patients with acute hemorrhagic stroke than in healthy control individuals [71]. Furthermore, in a case-control study, Yang et al. suggested that plasma copper was significantly associated with a higher risk of ischemic stroke but not of hemorrhagic stroke [72]. Undeniably, the contradictory results obtained from these clinical studies may have resulted from differences in adjustments for confounding factors such as the characteristics, specimen type, and study sample size of each observational study. Few studies have systematically assessed whether the association between copper and stroke varies on the basis of stroke subtype and stage. Therefore, it remains undetermined what the specific biologic mechanism is by which copper affects stroke. On one hand, copper proteins are essential in the cellular respiration of eukaryotic cells and are involved in the electron transfer processes of cellular respiration. As a cofactor in copper-dependent SOD and CP, Cu plays a vital role in numerous metabolic and oxidative reactions [73]. Cu/Zn-SOD1 is a dimeric cytoplasmic enzyme that detoxifies superoxide anion to H_2_O_2_. SOD1 overexpression reduces ROS levels and increases neural stem cell survival, which protects rodent brains from the damage of transient focal cerebral ischemia and transient global ischemia [74, 75]. In addition, Jiang, et al. found that the SOD1 nanozyme could rescue the brain in rats after stroke by locally protecting the cerebrovascular system [76].

On the other hand, copper can aggravate ischemic stroke [77]. It is well known that endothelial progenitor cells (EPCs) can promote angiogenesis and have been successfully used to restore endothelial function and enhance angiogenesis in ischemic brain tissue [78]. Thrombospondin-1 is a key inhibitor of EPC function [79]. Jiang et al. showed that copper can inhibit EPC function in copper-treated mice by increasing the level of thrombospondin-1, thereby aggravating ischemic stroke in mice [80]. Notably, in a recent clinical survey (3425 participants who were 20 years of age and older), investigators explored the association of serum copper with stroke risk factors (e.g., lipid levels) [81]. They found that serum copper was positively correlated with lipid levels in women, suggesting that copper may affect stroke by affecting lipid levels [81]. However, this remains to be confirmed with prospective studies.

### Ischemia/reperfusion injury

Ischemia/reperfusion injury refers to the damage sustained by tissues when the blood supply is restored after a period of ischemia or hypoxia [82]. After tissue ischemia, the increase in ROS and decrease in NO in activated endothelial cells lead to an increase in inflammatory factors such as interleukins and free radicals, resulting in an inflammatory response [83]. In some studies, low concentrations of copper ions exacerbated the degree of tissue ischemia/reperfusion injury, whereas administering an optimal level of copper ions ameliorated tissue damage. In rats, treatment with divalent copper ion significantly reduced malondialdehyde and myeloperoxidase activity while elevating glutathione content and SOD activity, thus protecting against tissue death by reducing free radical production and cell death [84]. In rat studies of liver ischemia/reperfusion, the use of the anti-ischemic agent mitragynine during ischemia/reperfusion elevated hepatic Cu/Zn-SOD levels, which reduced the occurrence of liver inflammation and injury. This suggested that copper ions also play a protective role in liver ischemia/reperfusion [85]. In addition, SOD reduced tissue damage induced by a free radical attack during intestinal ischemia/reperfusion in mice [86]. Sahu et al. showed that bromelain copper nanoparticles protect against associated ischemia/reperfusion-induced myocardial infarction [87]. A more recent study from the National Health and Nutrition Examination Survey also showed that increased dietary copper intake was negatively associated with the risk of myocardial infarction [88].

In summary, elevating the concentration of divalent copper ions within a certain range can reduce tissue damage during ischemia/reperfusion, whereas a high concentration of copper ions can induce cell death. It is worth mentioning that a consensus has not been reached regarding the optimal copper concentration for restoring ischemia/reperfusion injury. This may be due to different administration methods used in studies. In some cases, researchers used injections of copper-containing solutions and the oral administration of copper-containing nanoparticles to explore whether elevating the concentration of divalent copper ions reduces tissue damage during ischemia/reperfusion. In other cases, cells were directly exposed to copper salts to study copper-induced cell death. Differences in experimental results can also result from the different ion sensitivity of various organs. In the future, standardizing the experimental methods and increasing the cross-sectional comparison of different organs will be beneficial for the clinical application of copper ion modulator therapy.

### Heart failure (HF)

HF affects more than 64 million people worldwide, with an estimated prevalence of 1–2% in adults of developed countries. Most patients with HF have other comorbidities [89]. Failed heart muscle is metaphorically referred to as a “fuel-depleted engine.” If mitochondria are impaired in their ability to convert energy substrates into fuel (ATP), the burden on heart function increases. In mitochondria, copper is an important component of CCO, and copper deficiency leads to mitochondrial dysfunction [41]. Kang et al. showed that, in mice with HF, diet-induced copper deficiency for 5 weeks caused myocardial dysfunction [90]. Elsherif et al. went a step further to determine whether dietary copper deficiency–induced HF was reversible after copper supplementation [91]. They found that diastolic and systolic function and blunted responses to β-adrenergic stimulation were fully restored in copper-deficient mice fed with Cu supplements for 4 weeks [91]. This suggested that the alteration of β-adrenergic receptor (β-AR) signaling may be crucial for the pathogenesis of HF and that restoration of β-AR levels represents a possible therapeutic target [90].

Notably, in diabetes-induced HF, disordered myocardial copper transport was observed [92]. The hearts of patients with diabetes displayed impaired mitochondrial copper regulation, as reflected in decreased mRNA and/or protein levels and altered mitochondrial translocation of copper chaperone proteins, including COX17, COX11, and mitochondria-resident CCS. Interestingly, the copper chelator triethylenetetramine (TETA) restored copper transport in cardiomyocytes and significantly improved cardiac function [92]. TETA treatment restored the impaired structure and function of the myocardium in the hearts of patients with diabetes by restoring copper chaperone proteins and assembly factors for CCO. Trientine, a copper ion chelator, can also significantly improve cardiac function in animals with established HF [93]. However, the authors of this study did not explain the specific mechanism. We speculate that it may be related to the inhibition of Cu-mediated oxidative stress. Therefore, the comprehensive effect of chelating agents may be related to the etiology of heart failure. The principles underlying this relationship are worth exploring in the future.

In a meta-analysis that included data from 1504 individuals, a significant association was observed between high serum copper levels and HF [94]. An increased serum ratio of Cu/Zn is positively associated with lung cancer [95], aging [96], pneumonia [97], and ischemic heart disease [98]. Similarly, an increased serum Cu/Zn ratio is associated with an increased risk of HF in middle-aged and older Finnish men [99]. Elevated CP levels are associated with an increased risk of HF and poor prognosis in patients [100]. Whether the content of serum copper and myocardial copper maintains a proportional relationship in patients with HF warrants further consideration.

Overall, copper ions have been observed experimentally in different types of CVD, revealing their dual nature. Prospective cohort studies of copper intake in CVD have also shown inconsistent findings (Table 2), warranting more standardized and larger copper-related therapeutic research studies in patients with CVD. In the next section, we discuss the therapeutic strategies for targeting copper-induced cell death in CVD (Table 3).

**Table 2:** Prospective cohort studies of dietary copper intake in CVD.

Year	Location	Number of patients	Age (years)	Sex	Follow- up (years)	Disease	Substance of experiment	Findings	Ref.
1967	Finland	206	>31	Both	<1	CVD	High-sensitivity C-reactive protein (10 mg/l)	Copper is strongly associated with inflammatory burden	[138]
1989	Finland	2492	42–61	Male	5	CVD	Copper ion concentration 1.11 ± 0.18 mg/l	Association between high serum copper levels and increased risk of atherosclerotic CVD	[139]
1999	Japan	58,646	40–79	Both	10	CVD	The average copper intake of all patients is 1.33 ± 0.27 mg/day	Dietary copper intake is positively associated with cardiovascular mortality in men and women	[140]
2001	Finland	1866	42–61	Male	3	HF	Streptozotocin (55 mg/kg)	Increased serum Cu/Zn ratio is associated with increased HF risk in a linear dose-response fashion and may improve HF risk assessment	[141]
2010	Japan	196	80–98	Both	3	CVD	–	Decreased MCTs are associated with greater serum copper levels	[142]
2014	USA	1427	13–19	Both	3	CVD	111.7–129.7 μg/dl	Serum copper concentration correlates with total cholesterol concentration in adolescents	[143]
2018	USA	10,550	20–80	Both	5	CVD	The average copper intake of all patients is 2.19 ± 0.40 mg	The association between copper intake and stroke risk is more pronounced in female individuals and individuals younger than 65 years	[67]
2021	USA	30,899	>20	Both	11	CVD	Copper supplement (1.8 mg/day)	Copper-containing dietary supplement use is not associated with mortality in US adults	[144]

*CVD* cardiovascular diseases, *HF* heart failure, *MCT* mean cortical thickness.

**Table 3:** Potential therapies regulating copper in cuproptosis-related CVD.

Function	Copper drug	Ref.
Copper chelators, decrease the intracellular copper concentration	Tetrathiomolybdate	[101]
	Triethylenetetramine	[106]
	EDTA	[109]
	Trientine dihydrochloride	[112]
Natural antidote, more finely decreases the intracellular copper concentration	Turmeric	[117]
	Chalkophomycin	[119]
Inhibitor of copper chaperones, more specifically and finely reduces the intracellular copper concentration	DCAC50	[120]
Copper ionophore, transports copper into cells	8-hydroxyquinoline	[107]

*CVD* cardiovascular disease, *EDTA* ethylene diamine tetraacetic acid.

## Therapeutic strategies for targeting copper-induced cell death in CVD

### Copper chelators

Tetrathiomolybdate (TTM) is a small hydrophilic compound that chelates copper with high specificity. Currently, TTM has shown a favorable safety profile for treating Wilson’s disease, an autosomal recessive disorder characterized by excess copper accumulation in the liver [101]. TTM chelates bioavailable copper by forming a tripartite TTM-copper-protein complex. Alvarez et al. found that TTM specifically formed a complex with copper and its intracellular chaperone ATX1 by forming sulfur-bridged copper-molybdenum clusters [102]. The formation of this stable TTM-copper-ATX1 complex primarily contributed to the inhibition of copper delivery to the TGN and its downstream incorporation into cuproproteins. Wei et al. investigated the effect of TTM on inhibiting the development of atherosclerotic lesions in apolipoprotein E-deficient (*ApoE*^*−/−*^) mice [103]. They found that TTM inhibited atherosclerosis in *ApoE*^*−/−*^ mice by reducing bioavailable copper and vascular inflammation but not by altering iron homeostasis or reducing oxidative stress.

Triethylenetetramine (TETA) is a chelator that binds specifically and selectively to Cu^2+^ ions and has been used as a second-line treatment for Wilson’s disease [104, 105]. Yang et al. found that TETA inhibited the increase in serum copper levels and effectively abolished elevated CP activity after myocardial ischemia [106]. Zhang et al. revealed that TETA treatment improved myocardial function in the hearts of patients with diabetes by restoring mitochondrial CCO, mt-CCS, and mt-SOD1 activity [92].

The copper carrier 8-hydroxyquinoline and its derivatives can perform metal chelation, and they have a variety of biologic applications in different disease conditions ranging from neurodegenerative diseases to cancer [107]. In 2018, Yang et al. designed and synthesized a series of 8-hydroxyquinoline derivatives [108]. Compound 5b, a kind of 8-hydroxyquinoline derivative, was found to significantly inhibit metal (Cu^2+^ and Zn^2+^)-induced Aβ aggregation (88.9% for Cu^2+^, 73.3% for Zn^2+^). This finding suggests that compound 5b may be an alternative therapeutic for CVD induced by excessive iron.

Disodium ethylene diamine tetraacetic acid (EDTA) can chelate a wide range of metals, including copper [109]. Results of a 10-year clinical trial to assess chelation therapy showed that EDTA disodium-based infusion reduced recurrent cardiovascular events in patients with type 1 and type 2 diabetes who had previous myocardial infarction [110]. However, in a meta-analysis by Villarruz-Sulit et al. that included five studies with a total of 1993 randomized patients, chelation had no therapeutic effect on atherosclerotic vascular disease [111]. In three of the five studies, patients with peripheral vascular disease were recruited; in two studies, patients with coronary artery disease were recruited; and in one study, patients specifically with myocardial infarction were recruited. The conflicting outcomes from these studies may be attributed to differences in study design and patient populations.

Trientine dihydrochloride is a copper chelator approved for the treatment of Wilson’s disease. In an open-label pilot study, 20 patients with hypertrophic cardiomyopathy received trientine for 6 months [112]. Energy expenditure is hypothesized to be a major factor in the pathogenesis of hypertrophic cardiomyopathy. In this study, trientine selectively chelated Cu^2+^ to improve mitochondrial function and energy metabolism [112]. In addition, trientine reversed diabetes-induced mitochondrial ultrastructural damage and normalized the myocardial expression and enzymatic activity of proteins involved in energy metabolism [113].

On the other hand, metal chelators have some disadvantages. For example, metal chelators have been shown to redistribute heavy metals from other tissues to the brain, thereby increasing their neurotoxicity, resulting in the loss of essential metals, such as copper and zinc, and some serious side effects, such as liver toxicity [114]. Compared with traditional metal chelators, the natural antidote is readily available and affordable and has few side effects. For example, turmeric is an excellent chelating agent for metal ions (including Cu) and is the rhizome of the herb *Curcuma longa* [115]. The major component of turmeric is curcumin, which has antioxidant, antimicrobial, anti-inflammatory, antiviral, and anticarcinogenic properties. It has been used in traditional Chinese medicine to treat metabolic diseases and CVD [116]. In vitro and in vivo experiments have confirmed the effectiveness of turmeric in the treatment of CVD [117]. Although there is similar evidence from clinical trials [117], clinical studies with a longer intervention duration and specific endpoints to assess health outcomes are necessary to fully assess the long-term protective efficacy of turmeric. In addition, because methanobactin chelates excess copper ions, it has potential for treating acute Wilson’s disease or other neurodegenerative diseases caused by copper overload [118]. Recently, Gong et al. isolated a new copper chelator from Streptomyces sp. CB00271 and named it chalkophomycin [119]. Chalkophomycin and its ligands are expected to be small-molecule drugs with therapeutic effects on Wilson’s disease, neurodegenerative disease, or CVD that act by restoring copper homeostasis. In conclusion, considering the advantages of natural antidotes and the fact that CVD is a chronic disease requiring long-term cooperative treatment, further exploration in this area of research is warranted.

### Small-molecule inhibitors of copper chaperone proteins

The clinical use of copper ion chelators often leads to low copper ion concentrations, resulting in serious toxic side effects that disrupt other normal physiologic processes that require copper ions. In addition, copper chelators may non-specifically chelate other metal cations, resulting in further toxic side effects. From this perspective, developing drugs that specifically regulate the concentration and distribution of intracellular copper ions is important for achieving therapeutic effects while reducing toxic side effects. In 2015, Wang et al. showed that the compound DCAC50 specifically inhibited cancer cell proliferation without affecting the survival of normal cells by blocking the intracellular transport of copper ions and binding to the copper chaperone proteins ATOX1 and CCS [120]. Studies in which DCAC50’s mechanism of action was studied have shown that this compound inhibits the activity of Cu/Zn SOD1, which uses copper ions as cofactors, by interfering with copper ion transport and raising ROS levels while simultaneously affecting mitochondrial function and reducing ATP production. Notably, DCAC50 also sensitizes human and canine osteosarcoma cells to carboplatin chemotherapy [121]. These studies may guide the development of drugs for treating CVD.

In studies of atherosclerosis, ATOX1 is increased in the intima of atherosclerotic lesions in *ApoE*^*−/−*^ mice and localizes to the nucleus in pathologic conditions such as hypertensive and atherosclerotic vessels [122]. In inflammatory endothelial cells, ATOX1 binds to TNF-α receptor-associated factor 4 (TRAF4) in a Cu-dependent manner and promotes the production of ROS [123]. The ATOX1-TRAF4 axis is therefore a novel therapeutic target for vascular inflammatory diseases such as atherosclerosis.

CCS delivers Cu^+^ to the major cytosolic cuproenzyme SOD1 [124]. Hwang et al. showed a neuroprotective effect of targeting CCS after ischemic neuronal damage in the hippocampus of gerbils [125]. Moreover, Fukai et al. showed that CCS reduction impaired angiogenesis and wound healing while promoting the development of various types of CVD [126]. Further exploration of DCAC50 and other related compounds in the treatment of CVD is warranted.

### Copper ionophore

Current approaches to addressing copper deficiency rely on universal copper ionophores. Copper ionophores are small molecules that bind to copper and transport it into cells. Notably, the well-known copper ionophore elesclomol, which has selectivity for cancer cells, has been used in clinical trials as a treatment for patients with cancer. However, the mechanism of elesclomol’s selectivity is not known, and whether this selectivity can be manipulated for other copper ionophores to treat CVD warrants further investigation. Other copper ionophores have been summarized in a review by Oliveri [127]. Notably, there are two disadvantages to the versatility and non-targeted nature of conventional copper ion carriers. First, the copper transport process cannot be accurately regulated. In the case of excessive copper supplementation, Fenton-like chemotaxis may cause oxidative damage to biomolecules [128]. Second, inappropriate copper delivery may result in tissue-specific defects. Copper may accumulate and subsequently trigger oxidative damage in unwanted tissues when indiscriminate copper delivery is used to treat copper deficiencies in specific organs [129]. For this reason, Su et al. introduced the concept of targeted ion carrier–based metal supplements (TIMS), which is a method of transporting metals in a site-specific manner in organisms [130]. They designed and synthesized the N-acetylgalactosamine functionalized ionic carrier Gal-Cu (gtsm). Targeted Gal-Cu (gtsm) ion carriers transported more copper to the liver and the least copper to organs other than the usual non-targeted Cu (gtsm) ion carriers. This concept has opened new avenues for the application of metals in CVD medicine.

Another solution to addressing copper deficiency is the development of nano-drug delivery systems. Recently, Liu et al. developed a multifunctional nanocomposite that combines CuS photothermal therapy with anti-atherosclerotic chemotherapy to release drugs in the weakly acidic microenvironment of atherosclerotic inflammation. Thus, this nanocomposite enables precise drug delivery for treating atherosclerosis [131]. In summary, the research and development for a new generation of selective copper ionophores should focus on specificity and targeting.

## Conclusion and future perspectives

In recent years, research focused on understanding the function of copper-related cell death in cancers, CVD, and other diseases has received extensive attention. Copper ions drive the abnormal aggregation of lipoylated proteins, followed by the downregulation of Fe-S cluster protein expression, causing proteotoxic stress and, ultimately, cell death [8]. However, copper can also affect cell death in other ways, such as through ROS, ER, and inflammatory responses. Copper-induced cell death provides a link between oxidative stress and inflammation and inevitably plays an important role in the pathogenesis of CVD such as atherosclerosis, stroke, ischemia-reperfusion, and HF. Because copper ions can act as a double-edged sword in cells, the results of clinical studies are conflicting regarding the relationship between copper ion levels and the development of CVD. Therefore, more convincing clinical trials are needed in the future. In addition, different organs may have unique optimal copper ion concentrations. Exploring the optimal copper ion concentration in different organs will provide an important reference for how to optimize drug treatment with copper ions. When developing copper ion regulators, important future directions to consider are organ specificity, cell specificity, and well-controlled release. Although various studies have provided meaningful insights into copper-induced cell death, many unanswered questions remain, such as what the copper-induced cell death phenotype is, how the aggregation of fatty-acylated proteins induces a cascade of cell killing, and whether there are other important roles for copper in mitochondria. By answering these questions, we will gain a deeper understanding of how copper cell death-related human disease occurs so that we may continue to develop new therapeutic strategies for treating CVD.

## Data Availability

All data generated or analyzed during this study are included in this published article.
